# Effects of different magnetic stimulation paradigms on post-stroke upper limb function: a randomized controlled trial

**DOI:** 10.3389/fneur.2025.1683552

**Published:** 2025-10-17

**Authors:** Li Xu, Hong Luo, Lin Huang, Shuang Chen, Huifang Liu, Wei Cui

**Affiliations:** Department of Rehabilitation Medicine, Sichuan Provincial People's Hospital, School of Medicine, University of Electronic Science and Technology of China, Chengdu, China

**Keywords:** stroke, upper limb motor function, repetitive transcranial magnetic stimulation, repetitive peripheral magnetic stimulation, functional magnetic resonance imaging

## Abstract

**Background:**

Current evidence suggests that repetitive transcranial magnetic stimulation (rTMS), repetitive peripheral magnetic stimulation (rPMS), and their combined application can all enhance upper limb functional recovery after stroke. However, their comparative therapeutic profiles, including relative advantages and limitations, have not been systematically characterized.

**Objectives:**

To compare rTMS, rPMS, and combined protocols for post-stroke upper limb recovery, analyzing both functional outcomes and neural mechanisms to guide therapeutic selection.

**Methods:**

Fifty-one stroke patients were randomly divided into an rTMS group, rPMS group, or a combined group. Before and after 3 weeks of intervention, all patients were assessed with the Fugl-Meyer assessment for the upper limb (FMA-UL), the Thumb Localizing Test (TLT), modified Barthel index (MBI), and resting-state functional magnetic resonance imaging (rs-fMRI).

**Results:**

The ΔFMA-UL and ΔMBI scores of the combined group were significantly better than the rTMS group and rPMS group. The ΔTLT scores of the combined group and rPMS were significantly better than the rTMS group, but there was no statistically significant difference in ΔTLT scores between rPMS and the combined group. Compared to the rTMS group, the rPMS group showed increased amplitude of low-frequency fluctuation (ALFF) in the ipsilesional superior frontal gyrus, cerebellum_8 area, and contralesional cerebellum_crus1; the combined group showed increased ALFF in the ipsilesional cerebellum_8 area, superior medial frontal gyrus, and contralesional cerebellum_crus2 area. Compared with the rPMS group, the combined group showed increased ALFF in the ipsilesional paracentral lobule, supplementary motor area, precentral gyrus, and superior medial frontal gyrus.

**Conclusion:**

Compared with rTMS, rPMS has certain advantages in improving proprioception after stroke, and combination therapy improves both motor and proprioception. Therefore, combination therapy is recommended to better promote the recovery of brain and limb function.

**Clinical trial registration:**

http://chictr.org.cn, Identifier ChiCTR2200065871.

## 1 Introduction

Stroke is currently one of the main causes of disability (1). Approximately 70% of stroke survivors suffer from upper limb motor dysfunction, which causes serious obstacles to patients’ daily life, harms their physical and mental health, and heavily burdens their families and society (2). Repetitive transcranial magnetic stimulation (rTMS) is a non-invasive brain stimulation technique that modulates brain activity by releasing electromagnetic pulses through a coil placed on the subject’s head (3). It has been widely used in post-stroke rehabilitation treatment by directly regulating the plasticity of brain center (4), improving the sensorimotor system, and promoting the recovery of upper limb function after stroke (5). According to the rTMS guidelines, the application of low-frequency rTMS (LF-rTMS) in subacute hand dysfunction after stroke is highly recommended and has definite therapeutic effects (4).

Repetitive peripheral magnetic stimulation (rPMS) is a non-invasive treatment method that directly or indirectly activates peripheral motor nerves, generates action potentials of motor neurons, causes muscle contraction, and has the advantage of painless extension to deeper muscle areas (6). Moreover, rPMS can also avoid adverse reactions caused by rTMS, such as dizziness and scalp discomfort, and can be applied to patients with metal head implants. In musculoskeletal or nervous system diseases, rPMS may have different effects on neuroplasticity involved in pain relief and motor recovery and is considered to be a promising and easy-to-manage neuromodulation technique for motor recovery after stroke (7, 8). Obayashi et al. (9) found that rPMS improved severe upper limb paralysis in early acute stroke survivors; they found significant improvement in the upper limb motor component of the Fugl-Meyer motor assessment (FMA-UL) and the Wolf motor function test (WMFT) after upper limb rPMS treatment. Jiang et al. (10) found that rPMS of the upper limb extensor muscles can promote upper limb arm function and grip strength as well as muscle strength for elbow flexion and extension. However, it is still unclear whether rPMS can achieve the same effect as rTMS in upper limb dysfunction after stroke, and further exploration is needed.

In recent years, some researchers have proposed that rTMS combined with rPMS may have better therapeutic effects than single magnetic stimulation (11). Qin et al. (12) found that LF-rTMS combined with rPMS could produce better improvement in upper limb motor function and spasticity than rTMS or conventional rehabilitation treatment alone and believed that the better results may be related to the changes in the activity of the cerebellum and frontoparietal cortex. Some researchers have also found that upper limb rPMS may have a synergistic effect on central intermittent theta-burst stimulation (iTBS), thereby improving grasping function (6). Although studies have demonstrated that the combination of rTMS and rPMS can effectively promote the recovery of upper limb motor function after stroke, research on this combined therapy remains limited. Whether the combination is superior to single rTMS or rPMS requires further investigation, and its underlying mechanisms remain unclear.

Resting-state functional magnetic resonance imaging (rs-fMRI) measures changes in blood-oxygen-level-dependent (BOLD) signal to observe the intrinsic functional activity or connectivity of the brain in the resting state (13). Due to its non-invasive, non-radiative, and high spatiotemporal resolution characteristics, rs-fMRI can display the functional activity of the entire brain network and is currently widely used in brain function research. The amplitude of low-frequency fluctuation (ALFF) can reflect the strength of the spontaneous activity level of each voxel neuron from an energy perspective, which is used to characterize the local properties of rs-fMRI signals (14). Previous studies have shown that post-stroke motor recovery outcomes had a strong correlation with ALFF values, indicating that ALFF may have potential as a prognostic biomarker for post-stroke motor recovery (15, 16).

Based on this, we designed a single blind randomized controlled clinical trial to compare the effects of low-frequency rTMS, rPMS, and rTMS combined with rPMS on the recovery of upper limb motor and sensory functions in patients with subacute stroke. At the same time, rs-fMRI was used to analyze the functional brain areas of the stroke patients and observe changes in brain plasticity under different magnetic stimulation methods.

## 2 Materials and methods

### 2.1 Study design

This study is a single-blind (evaluator) randomized controlled trial. The effect size was calculated as 0.19, based on the change in FMA-UL scores observed in the pilot test. With 80% statistical power and an *α* level of 0.05, the three groups required at least 15 patients each. Considering a drop-out rate of 10%, 17 patients were included in each group. This study was approved by the Medical Ethics Committee of Sichuan Provincial People’s Hospital, and the number was 2022–349. The clinical trial number is ChiCTR2200065871. A computer-generated randomization list was generated by a research assistant who did not participate in the experiment. Each random permutation was transferred into a series of consecutively numbered, sealed, and opaque envelopes. The evaluators were blinded to treatment assignments until the end of the study. All patients were evaluated before and 3 weeks after the intervention.

### 2.2 Participants

**Figure 1:**
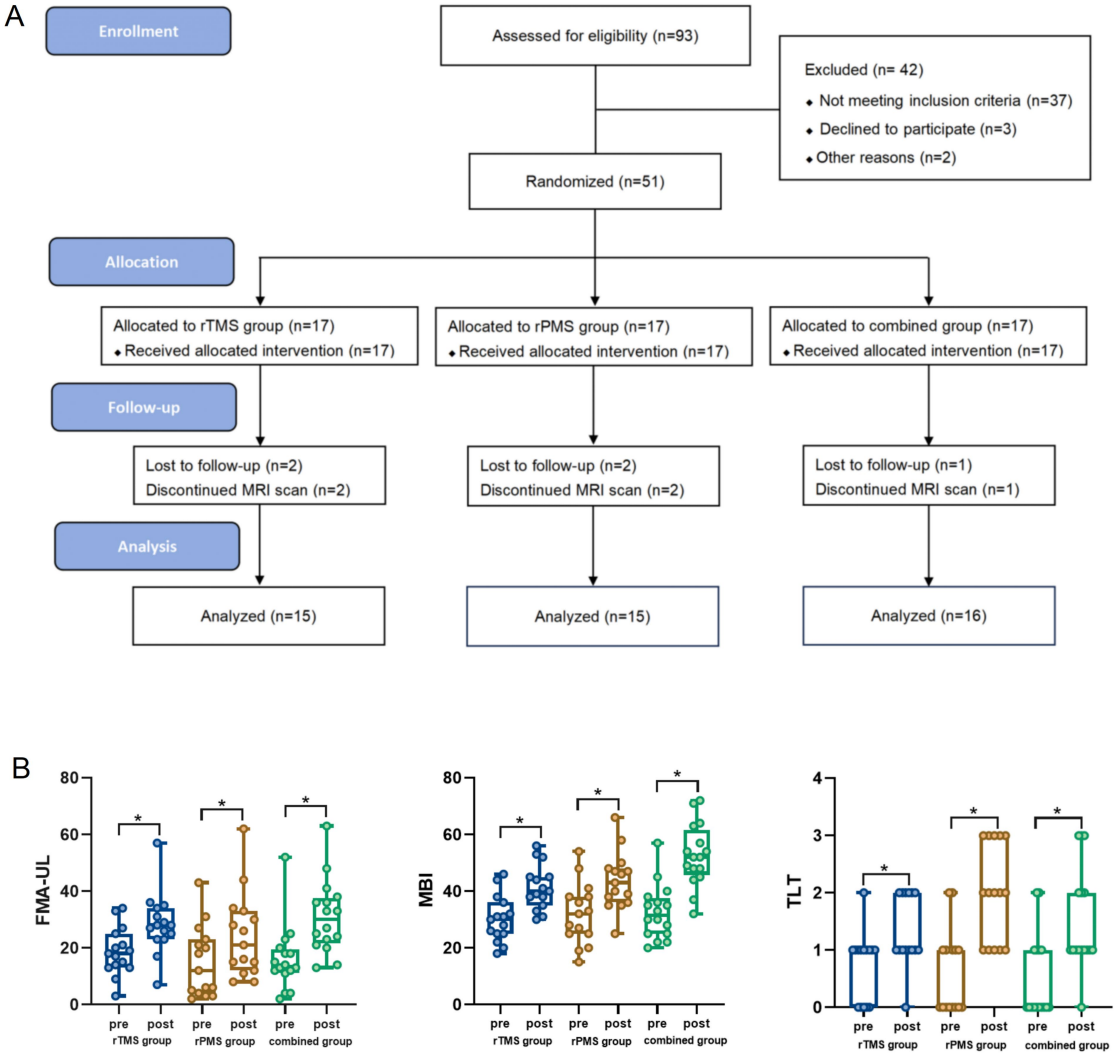
**(A)** Flow diagram; **(B)** the FMA-UL, MBI, and TLT scores before and after treatment of the three groups. Error bars indicate standard deviation of the mean. *p < 0.05. FMA-UL, Fugl-Meyer assessment for the upper limb; MBI, modified Barthel Index. TLT, Thumb Localizing test.

From November 2022 to November 2023, 51 stroke patients hospitalized in the department of rehabilitation were selected as the participants. Ultimately, 46 patients were recruited in this study, and they were divided into the central group (rTMS group, *n* = 15), peripheral group (rPMS group, *n* = 15), or rTMS combined rPMS group (combined group, *n* = 16; Figure 1A).

(1) All participants had to meet the following criteria: ① meet the diagnostic criteria of stroke revised by the diagnostic criteria of cerebrovascular diseases in China (version 2019) (17) and have it confirmed by transcranial CT or MRI that the responsible lesion is in the unilateral basal nucleus and/or radiation coronal region; ② aged 25–75 years; ③ first onset, with a course of less than 3 months; ④ no contraindication of MRI examination; ⑤ right handed; ⑥ conscious and with stable vital signs; ⑦ no severe cognitive impairment based on the Mini-Mental State Examination (MMSE) score ≥ 17, primary school level ≥ 20, middle school level (including technical secondary school) ≥ 24; ⑧ the motor-evoked potential(MEP) in the primary motor area (M1) of the affected side of the patient could be detected; and ⑨ informed consent was signed by the patient or legal guardian.
(2) The exclusion criteria consisted of the following: ① experience of craniotomy; ② multiple strokes; ③ previous history of epilepsy, obvious intellectual disability, dementia, etc., meaning the patient could not cooperate with the curative effect evaluation or MRI examination; ④ severe functional failure of important organs or hemorrhagic diseases and malignant tumors that would seriously affect the treatment process; or ⑤ pacemaker, cochlear implant, or metal or other objects implanted in the body.

The general data of the three groups were statistically compared, and there was no statistical difference between the groups (*p* > 0.05; Table 1).

**Table 1:** Demographic and clinical characteristics at baseline.

Characteristic	rTMS group (*n* = 15)	rPMS group (*n* = 15)	Combined group (*n* = 16)	*p* value
Age (years, mean ± SD)	55.80 ± 14.0	53.60 ± 16.45	56.40 ± 13.83	0.884
Gender (M/F)	10/5	10/6	11/5	0.806
Stroke type (I/H)	9/6	8/7	9/7	0.184
Time since stroke onset (days)	48.73 ± 6.13	45.09 ± 7.20	47.83 ± 8.26	0.482
FMA-UL	18.73 ± 8.47	14.93 ± 12.44	16.00 ± 11.58	0.622
MBI	30.13 ± 8.17	31.76 ± 10.35	32.56 ± 9.62	0.770
TLT	0.90 ± 0.56	0.60 ± 0.69	0.40 ± 0.69	0.250

M, Male; F, Female; I, Infarction; H, Hemorrhage; FMA-UL, Fugl-Meyer assessment for the upper limb; MBI, modified Barthel Index. TLT, Thumb Localizing test. SD, standard deviation.

### 2.3 Interventions

**Figure 2:**
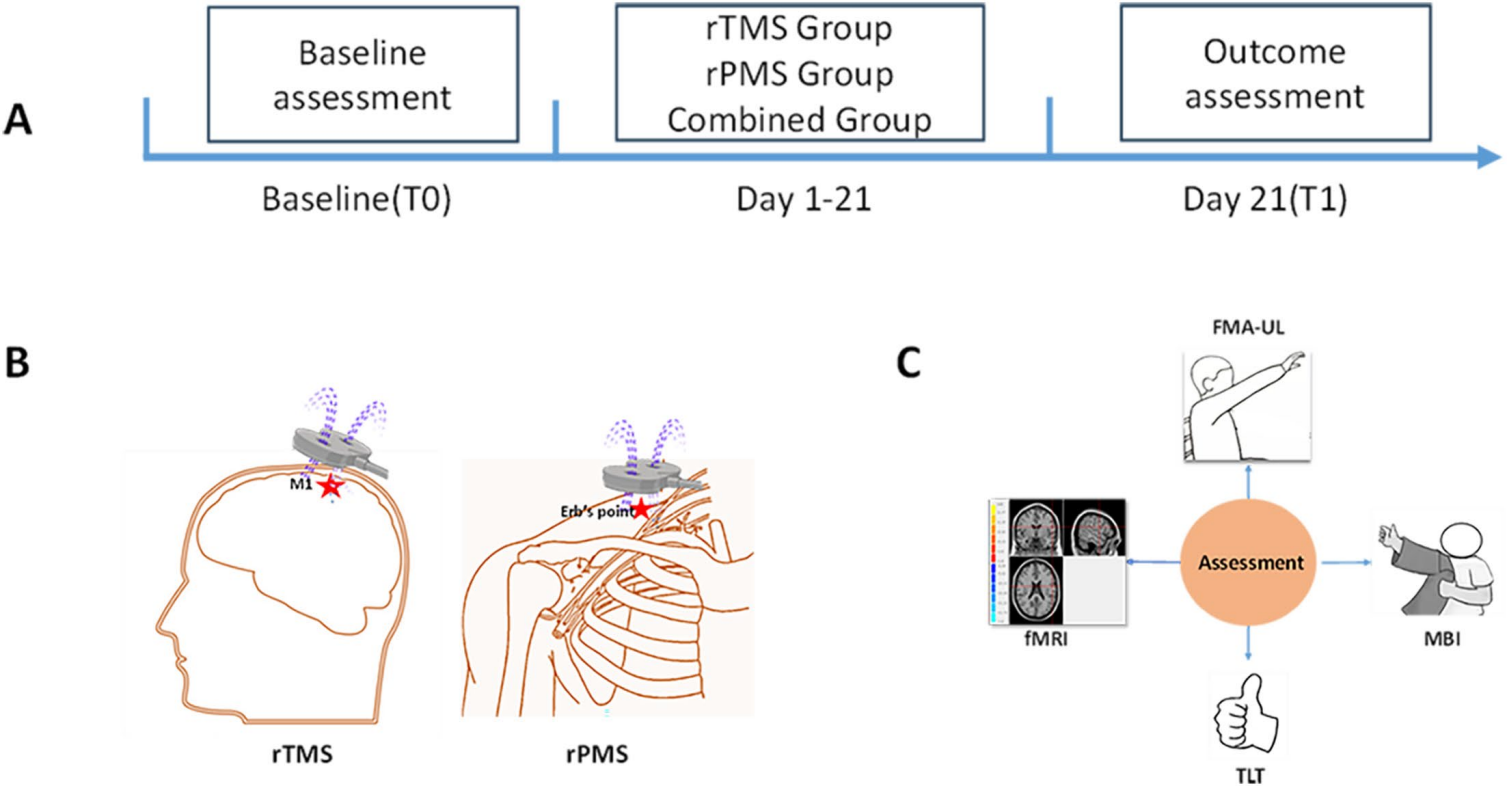
The experimental flow. **(A)** Timeline of assessment and intervention. At baseline (T0), clinical assessment and fMRI were used to evaluate the patients. Following 3 weeks of rTMS, rPMS or combined intervention, depending on group allocation, assessments are repeated post-treatment (T1). **(B)** Diagram of rTMS and rPMS interventions. **(C)** Assessment methods.

We selected the CCY-II transcranial magnetic stimulator made by Wuhan Yiruide Company and an eight-shaped coil for magnetic stimulation treatment. During the treatment, the patient remained in a stable supine position, and the center of the coil was placed at the target stimulation point. In the rTMS group, the magnetic stimulation coil was tangent to the skull surface, and 1 Hz LF-rTMS was applied in the unaffected M1 area for a duration of 10s, an interval of 4 s, and 860 pulses. The magnetic intensity was set at 100% of the resting motor threshold (RMT). RMT was defined as the minimum magnetic stimulation intensity that induced MEPs of ≥ 50 μV in the abductor pollicis brevis muscle in at least five out of 10 consecutive stimulations applied to the unaffected M1 (18). In the rPMS group, the affected upper limb was subjected to 5 Hz rPMS at the Erb’s point of the brachial plexus (19), each time lasting for 1.2 s, with an interval of 3 s, and 1716 pulses. The rPMS protocol used the same eight-shaped coil as rTMS in this study. The stimulation intensity for rPMS was individually adjusted for each participant to cause visible muscle contractions in the upper limb. The combined group performed rPMS for 10 min and then performed rTMS for 10 min. The unaffected M1 area received 430 LF-rTMS pulses at 1 Hz stimulation, and the affected side Erb’s point received 858 rPMS pulses at 5 Hz stimulation. The total treatment time was 20 min in all groups. All the patients in the three groups received magnetic stimulation once a day before physical therapy, which lasted for 3 weeks, 5 days a week, with an interval of 2 days. Other routine rehabilitation treatments were the same, including physical therapy, occupational therapy, acupuncture, and so on. The experimental flow is shown in Figure 2.

### 2.4 Clinical assessment

(1) Primary outcome measure.

The Fugl-Meyer assessment for the upper limb (FMA-UL): As the main evaluation result, each item was scored from 0 to 2, with a total score of 66 points. Higher scores indicated better limb motor function, and FMA-UL < 31 was classified as severe upper limb dysfunction (20).

The Thumb Localizing Test (TLT): Proprioception assessment was performed using TLT. The examiner moved the subject’s stroke-affected limb to a random position above eye level with the subject blindfolded. Subjects were instructed to use the opposite arm to grasp the thumb of the stroke-affected limb. The TLT is graded on a four-point scale from zero (no impairment) to three (unable to locate thumb) (21).

(2) Secondary outcome measure.

The modified Barthel index (MBI): The MBI is divided into 10 items, with a total score of 100. The lower the score, the more unable the patient is to take care of themselves in daily life, with 0–20 indicating extremely severe dependence. 25–40 denotes severe dependence; 45–60 denotes moderate dependence; 65–95 is mild dependence; and 100 relates to complete self-care (22).

(3) Resting-state fMRI acquisition.

A Siemens GET 3.0 T magnetic resonance scanner was used to scan the resting-state functional images of the patients in this study before and after magnetic stimulation treatment. The rs-fMRI images were acquired via a gradient- echo- planar imaging (EPI) sequence with the following parameters: repetition time (TR) = 2000 ms, echo time (TE) = 13 ms, field of view (FOV) = 192 × 192 mm^2^, flip angle (FA) = 90^°^, slice thickness = 3 mm, slice gap = 1 mm, matrix size = 64 × 64, and voxel size of 3 × 3 × 3 mm^3^. A total of 250 timing slices on axial view was obtained and the scanning time was 8′24″. Additional T1-weighted structural images were obtained by rapid acquisition gradient echo imaging sequence using the following parameters: TR = 1900 ms, TE = 2.52 ms, FA = 9^°^, slice thickness = 1.0 mm, Slice Gap = 0 mm, matrix size = 256 × 256, FOV = 250 × 250 mm^2^, and voxel size = 1 × 1 × 1 mm^3^. In total, 176 images were obtained, and the scanning time was 4′18″. When scanning, the patient was in a supine position, and the gap between the head and the coil was filled with a sponge pad to fix it, so as to reduce the influence of head movement during scanning. The patient was asked to close their eyes and relax quietly during testing.

### 2.5 Statistical analysis

(1) Clinical data analysis.

Statistical analysis was conducted using SPSS software (IBM SPSS Statistical Window, version 21.0, Armonk, NY). All data were checked for normality with the Shapiro–Wilk test. Two-way repeated measures were conducted on the variances that conformed to a normal distribution. Group (rTMS group, rPMS group, and combined group) and time (pre-treatment and post-treatment) were entered as fixed factors. For data that were not normally distributed, the Wilcoxon rank sum test and the Kruskal-Wallis test were used for within-group (pre- vs. post-treatment) and between-group comparisons, respectively. Functional recovery value (defined as the difference in scale values before and after magnetic stimulation) was used to compare the treatment effects. A one-way ANOVA test was used to compare the recovery values among different groups. *p* < 0.05 was considered statistically significant.

(2) fMRI data processing and analysis.

The images of patients with right lesions were flipped relative to the sagittal plane, so that the affected hemisphere of all patients was the left hemisphere. The data preprocessing was carried out using the Metlab2018a platform, and Dpass5.3 was used to preprocess three sets of data (before and after). The preprocessing process included temporal correction, head motion correction, combined structural image registration, standardization, and spatial smoothing. DEPASF software was used to remove interference, including regressing 24 head movement parameters, whole brain mean signals, and white matter signals, and to perform ideal bandpass filtering in the frequency range of 0.01–0.08 Hz. SPM12 paired with a sample t-test was used to test for differences in ALFF values before and after treatment, with gender, age, and years of education as covariates to reduce their potential impact. Single factor ANCOVA was conducted using RESTplus software to compare the differences in ALFF images among the combined group, rTMS group, and rPMS group. Subsequently, post-hoc analysis was used to compare the differences between groups. All statistics were adjusted for multiple comparisons using Alphasim values with *p* < 0.05, and Cluster≥ 16 were considered as significant regions.

## 3 Results

### 3.1 Clinical outcomes

Before treatment, there was no significant difference in FMA-UL, MBI, or TLT scores among the three groups (*p* > 0.05). After 3 weeks of treatment, the FMA-UL, MBI, and TLT scores of the three groups improved compared to before treatment (*p* < 0.05; Table 2; Figure 1B). The changes in FMA-UL and MBI scores in the combined group were significantly higher than those in the rPMS and rTMS groups (*p* < 0.05). The changes in TLT scores in the combined and rPMS groups were significantly higher than those in the rTMS group (*p* < 0.05); there was no significant difference in changes to TLT score between the combined group and the rPMS group (*p* > 0.05; Table 3).

**Table 2:** Intra- and intergroup comparison of clinical outcomes.

Variable	rTMS group (*n* = 15)	rPMS group (*n* = 15)	Combined group (*n* = 16)	*F*/χ ^2^ value (*Df*)	*p* value
FMA-UL Pre-treatment	18.73 ± 8.47	14.93 ± 12.44	16.00 ± 11.58	0.480(2)	0.622^a^
Post-treatment	28.40 ± 10.76	24.20 ± 14.97	30.94 ± 8.47	1.053(2)	0.358^a^
*p* value	0.001^a^	0.001^a^	0.001^a^		
MBI Pre-treatment	30.13 ± 8.17	31.76 ± 10.35	32.56 ± 9.62	0.263(2)	0.770^a^
Post-treatment	41.33 ± 7.89	43.53 ± 10.06	52.63 ± 11.05	5.844(2)	0.006^a^
*p* value	0.001^a^	0.001^a^	0.001^a^		
TLT Pre-treatment	0.80 ± 0.561	0.6 ± 0.737	0.50 ± 0.730	2.733(2)	0.298^b^
Post-treatment	1.27 ± 0.704	2.00 ± 0.845	1.50 ± 0.894	4.867(2)	0.077^b^
*p* value	0.003^c^	0.002^c^	0.001^c^		

Continuous data were expressed as mean **±** standard deviation. FMA-UL, Fugl-Meyer assessment for the upper limb; MBI, modified Barthel Index; TLT, Thumb Localizing test. Df, degrees of freedom.

a Two-way repeated measures ANOVA test.

b Kruskal-Wallis test.

c Wilcoxon rank sum test.

**Table 3:** Intergroup comparison of ΔFMA, ΔMBI, and ΔLTL scores among three groups.

Variable	rTMS group (*n* = 15)	rPMS group (*n* = 1 5)	Combined group (*n* = 16)	*p* value	*F* value (*Df*)	rTMS group vs. Combined group; *p* value	rTMS group vs. rPMS group; *p* value	rPMS group vs. Combined group; *p* value
ΔFMA	9.67 ± 5.32	9.33 ± 4.77	14.94 ± 6.83	0.014	4.692(2)	0.014	0.874	0.009
ΔMBI	11.20 ± 4.67	10.93 ± 2.40	20.06 ± 9.33	0.012	10.722(2)	0.019	0.075	0.009
ΔTLT	0.40 ± 0.56	1.40 ± 0.51	1.00 ± 0.66	0.002	9.226(2)	0.026	0.001	0.129

Continuous data were expressed as mean **±** standard deviation. FMA-UL, Fugl-Meyer assessment for the upper limb; MBI, modified Barthel Index. TLT, Thumb Localizing test. Df, degrees of freedom.

### 3.2 Rs-fMRI results

(1) Intragroup comparison

**Figure 3:**
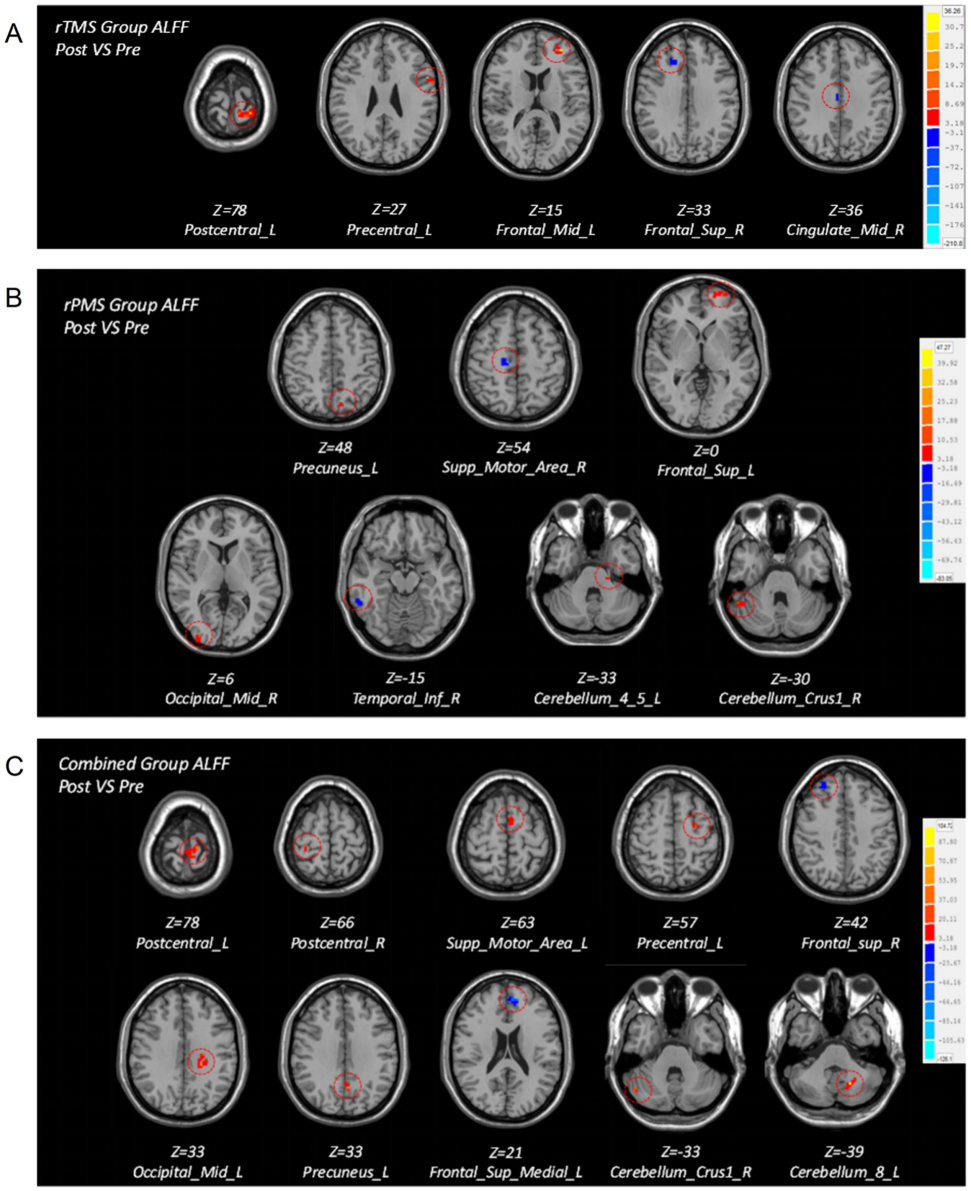
Brain maps of intragroup differences in ALF*F* values before and after treatment in the three groups. **(A)** Brain regions with differences in ALFF before and after treatment in the rTMS group. **(B)** Brain regions with differences in ALFF before and after treatment in the rPMS group. **(C)** Brain regions with differences in ALFF before and after treatment in the combined group.

After treatment, the rTMS group showed higher ALFF in the ipsilesional precentral gyrus, postcentral gyrus, and middle frontal gyrus and lower ALFF in the contralateral superior frontal gyrus, median cingulate, and paracingulate gyri. After treatment, the rPMS group patients showed higher ALFF in the ipsilesional superior frontal gyrus, precuneus gyrus, cerebelum_4_5 gyrus, contralateral middle occipital gyrus, and cerebellum_crus1 and lower ALFF in the contralateral supplementary motor area (SMA) and inferior temporal gyrus. After treatment, the combined group patients showed higher ALFF in the ipsilesional cerebelum_8 area, middle occipital gyrus, postcentral gyrus, precentral gyrus, precuneus gyrus, SMA, and contralateral cerebellum_crus1 area and lower ALFF in the contralateral superior frontal gyrus and ipsilesional superior medial frontal gyrus (Table 4; Figure 3).

(2) Group comparison

**Figure 4:**
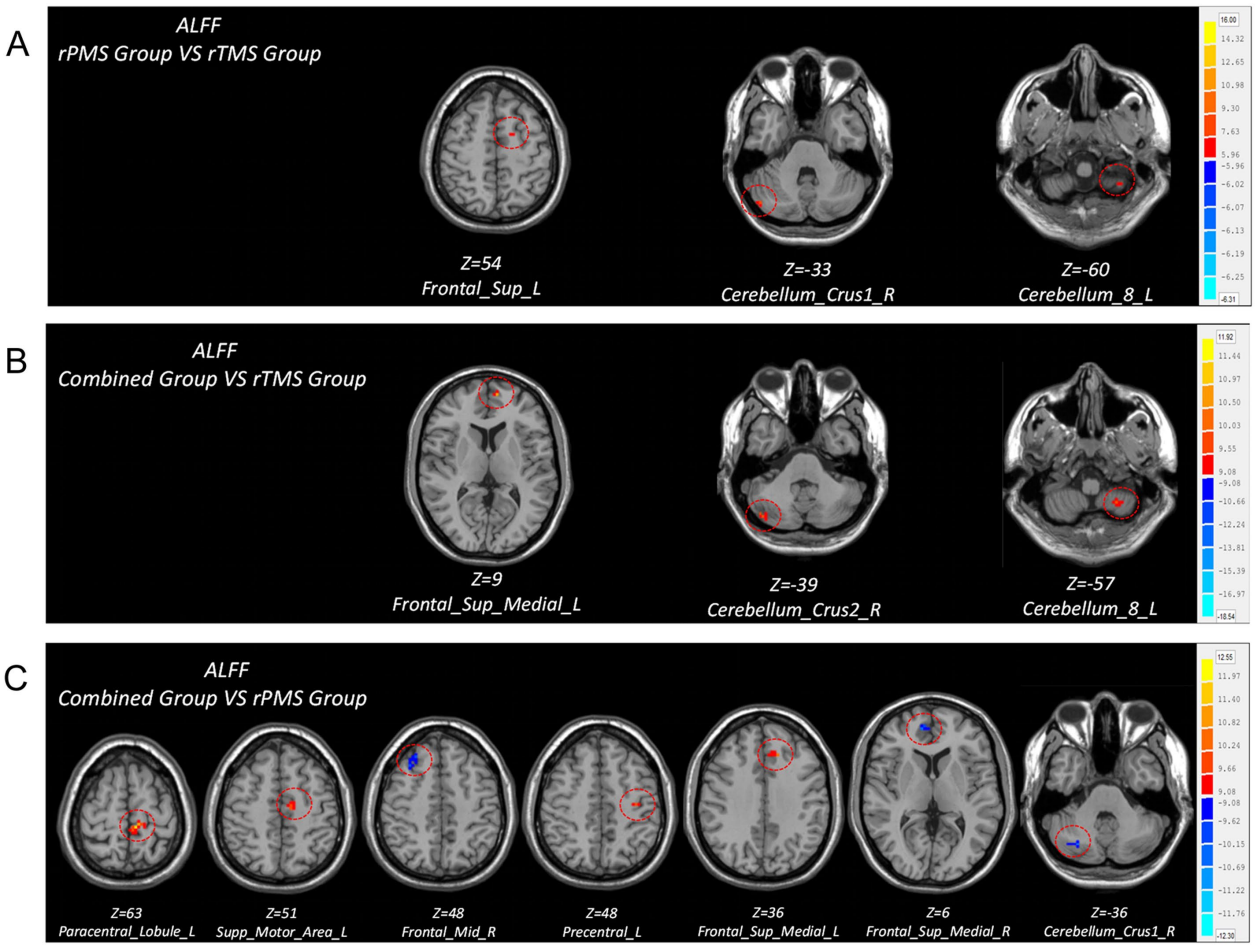
Brain regions with differences in ALFF values after treatment among three groups. **(A)** Comparison of ALFF differences between rPMS group and rTMS group. **(B)** Comparison of ALFF differences between combined group and rTMS group. **(C)** Comparison of ALFF differences between combined group and rPMS group.

Compared to the rTMS group, the post-hoc analyses revealed that the rPMS group showed increased ALFF in the ipsilesional superior frontal gyrus, cerebellum_8 area, and contralesional cerebellum_crus1 area post-intervention; the combined group showed increased ALFF in the ipsilesional cerebellum_8 area, superior medial frontal gyrus, and contralesional cerebellum_crus2 area post-intervention. Compared to the rPMS group, the combined group showed increased ALFF in the ipsilesional paracentral lobule, SMA, precentral gyrus, and superior medial frontal gyrus and decreased ALFF in the contralesional cerebellum_crus1 area, superior medial frontal gyrus, and middle frontal gyrus post-intervention (Table 5; Figure 4).

**Table 4:** Brain regions with significant differences in ALFF between the three groups.

ALFF	Brain regions	MNI coordinate			Peak T value
		X	Y	Z	
ALFF increase in rTMS group	Frontal_Mid_L	−33	57	15	32.4781
	Postcentral_L	−18	−39	78	23.426
	Precentral_L	−60	9	27	9.4157
ALFF decrease in rTMS group	Frontal_Sup_R	21	30	33	−9.7434
	Cingulate_Mid_R	6	−15	36	−13.3513
ALFF increase in rPMS group	Cerebelum_4_5_L	−21	−30	−33	10.1156
	Frontal_Sup_L	−21	63	0	14.6966
	Occipital_Mid_R	39	−93	6	8.7635
	Precuneus_L	−12	−66	48	27.8871
	Cerebellum_Crus1_R	54	−48	−30	10.5585
ALFF decrease in rPMS group	Supp_Motor_Area_R	6	−27	54	−16.8921
	Temporal_Inf_R	63	−48	−15	−11.4667
ALFF increase in Combined group	Cerebellum_8_L	−24	−60	−39	104.7201
	Occipital_Mid_L	−27	−57	33	62.2626
	Postcentral_L	−21	−39	78	42.7427
	Postcentral_R	30	−33	66	29.2757
	Precentral_L	−27	−12	57	23.9396
	Precuneus_L	−3	−57	33	38.2564
	Supp_Motor_Area_L	−3	3	63	35.3665
	Cerebellum_Crus1_R	54	−69	−33	20.8879
ALFF decrease in Combined group	Frontal_Sup_Medial_L	−6	48	21	−52.4302
	Frontal_Sup_R	24	42	42	−17.1548

ALFF, amplitude of frequency fluctuation; MNI, the Montreal Neurological Institute. All displayed brain regions showed *p* < 0.05.

**Table 5:** Brain regions with significant differences in ALFF among the three groups.

ALFF	Brain regions	MNI coordinate			Peak T value
		X	Y	Z	
rPMS group vs. rTMS group	Cerebelum_8_L	−33	−54	−60	3.6447
	Frontal_Sup_L	−18	0	54	3.9911
	Cerebellum_Crus1_R	48	−75	−33	5.4166
Combined group vs. rTMS group	Cerebellum_8_L	−27	−54	−57	4.3405
	Frontal_Sup_Medial_L	−6	57	9	8.5213
	Cerebelum_Crus2_R	45	−78	−39	5.071
Combined group vs. rPMS group	Frontal_Sup_Medial_L	−9	30	36	8.4953
	Paracentral_Lobule_L	−6	−33	63	10.4522
	Precentral_L	−36	−18	48	5.3288
	Supp_Motor_Area_L	−9	−12	51	5.2142
	Cerebelum_Crus1_R	27	−72	−36	−5.9325
	Frontal_Sup_Medial_R	9	51	6	−6.2325
	Frontal_Mid_R	30	33	48	−4.3268

ALFF, amplitude of frequency fluctuation; MNI, the Montreal Neurological Institute. All displayed brain regions showed *p* < 0.05.

## 4 Discussion

This study systematically compared the effectiveness of rTMS, rPMS, and their combined application in restoring post-stroke upper limb motor function to determine their relative merits. The results showed that the motor function of the three groups after treatment had different degrees of improvement compared to before treatment. Compared to rTMS, the rPMS group showed no significant change in FMA-UL scores, but there was a significant improvement in proprioception- related score ΔTLT. The improvement of FMA-UL score in the combined group was higher than that in the rTMS group and rPMS group, and the proprioceptive change (ΔTLT) in the combined group was better than that in the rTMS group, but there was no significant difference between the combined group and the rPMS group. Based on the above results, we believe that rPMS and rTMS have comparable effects on promoting upper limb motor recovery while combined treatment has better advantages in improving motor function, which is consistent with the existing research results (23). Combined with changes in proprioception assessment results, we also believe that rPMS has a unique advantage in improving patients’ proprioception.

RPMS is believed to activate deep conductive structures and generate strong muscle contractions and substantial proprioceptive inputs while exhibiting minimal skin recruitment, which can significantly improve sensory and motor impairments caused by brain injury (24, 25). In this study, there was no significant difference in *Δ* FMA-UL changes between the rPMS group and the rTMS group, indicating that rPMS and rTMS have the same promoting effect on the recovery of upper limb motor dysfunction after stroke. This is similar to the research results of scholars such as Kamo, Obayashi, and Jiang, who all found that rPMS can significantly improve the recovery of upper limb motor function after stroke (7, 9, 10). In the pairwise comparisons of ALFF values between groups, we found that the group involving rPMS significantly enhanced the activation response in the contralateral cerebellar crus region. The cerebellar crus area critically modulates upper limb and hand proprioception, coordinates with the primary motor cortex (M1) and premotor cortex (PM), and contributes to the execution of complex motor tasks (26). Animal experiments have shown that the crus region has a close spatial correspondence with the primary somatosensory cortex (SI) region. This has led to the proposed coherent topographic organization of the cerebro-ponto-cerebellar networks, verifying the brain functional connection between somatosensory perception and the cerebellum (27). The cerebellum gains proprioceptive afferents from various receptors mainly through the tractus spinocerebellar, and previous positron emission tomography and fMRI studies demonstrated that there was widespread activation of the cerebellum during active and passive movements (28). The cerebellum is involved in the discrimination and integration of various sensory inputs, enabling the integration of sensory-motor information to form an internal model within the cerebellum, which can predict the sensory consequences of behavior (29). These findings suggest that the cerebellum plays an important role in proprioception (30). Based on the significant improvement in proprioceptive scores observed in the group treated with rPMS in this study, we speculate that the cerebellum is one of the key brain regions significantly affected by rPMS intervention. Studies have shown that rPMS can directly stimulate Ia sensory fibers or induce repeated muscle/joint contractions through magnetic pulses, indirectly producing a large amount of proprioceptive inputs (31). We speculate that this proprioceptive stimulus can directly stimulate sensory motor input nerve fibers through forward and backward conduction, inducing nerve fibers to project to the corresponding spinal nerves and extraspinal systems, promoting spontaneous neural activity in the cerebellum, and strengthening its functional connection with other brain regions, thus playing a positive role in the recovery of proprioceptive sensation and motor function. Therefore, we believe that, although rPMS treatment stimulates at the distal end, it still has a significant regulatory effect on the cerebral cortex. This is consistent with the results of several studies suggesting that rPMS induces proprioceptive inflows that affect motor planning mechanisms at the cortical level (32–35). This is also consistent with the findings of Gardoni et al., which indicated that increased activation of crus I is associated with better motor performance (36).

The improvement of FMA-UL score in the combined group was higher than that in the rTMS and rPMS group, and the proprioceptive changes in the combination group were better than those in the rTMS group, which proved that rPMS combined with rTMS has a stronger synergistic effect. In this study, LF-rTMS was used to directly inhibit the contralateral M1 area to regulate cortical activity, thereby promoting the balance of excitability between the hemispheres and inducing plasticity changes. High-frequency magnetic stimulation of peripheral neuromuscular by rPMS induces muscle contraction and increases proprioceptive input from peripheral limbs to the central nervous system, promotes motor output modulation, and improves sensorimotor integration (37, 38). The combined therapy promotes central nervous system reorganization through both peripheral and central mechanisms. The results of our study are consistent with the results of Qin and Wu et al., who found that rTMS combined with rPMS can promote the recovery of upper limb motor function better than single treatment (12, 39). Our study found that the combined group showed statistically significant enhancement in the ALFF values of the main sensory and motor areas, such as the cerebelun_crus area, precentral gyrus, SMA, and paracentral lobule. This is partially consistent with the findings of Qin et al. (12), whose study on combination therapy also found an increase in ALFF values in the SMA region after treatment, indicating that the combination of rTMS and rPMS can promote the reorganization of relevant motor areas after stroke. Kumru et al. (40)found that combined peripheral and central magnetic stimulation increased motor-evoked potentials amplitude of the extensor carpi radialis muscle and reduced short intracortical inhibition compared with rTMS or rPMS alone, indicating that combined therapy could increase corticospinal excitability and reduce intracortical inhibition. Gao et al. (41)found in the study of a rat model of middle cerebral artery occlusion that central combined with peripheral magnetic stimulation can significantly activate brain activity in the ipsilateral sensorimotor cortex, upregulate the expression of plasticity-related proteins in the brain, increase local brain activity, and promote functional recovery of the affected sensorimotor, ultimately altering behavioral recovery. The combination of rTMS and rPMS may form a circuit that can achieve excitation of the entire sensorimotor circuit, modulate the excitability of the relevant motor cortex, and facilitate functional reorganization of the cerebral cortex to restore normal activity patterns (42). We speculate that the combination of rTMS and rPMS can directly act on the motor cortex through rTMS, regulate cortical excitability, and reduce the interhemisphereal inhibition imposed on the affected side. Recruiting muscles and joint afferent nerves through rPMS generates greater proprioceptive influx, and the bottom-up sensory conduction system activates the motor cortex, generating positive feedback information input into the central nervous system (43). Combined magnetic stimulation may be a better magnetic stimulation treatment option for upper limb motor function recovery in stroke patients.

In summary, we concluded that both rPMS and peripheral combined central magnetic stimulation therapy offer similar motor function recovery effects to rTMS, indicating that both top-down and bottom-up magnetic stimulation can promote motor function recovery after stroke. The combined therapy has a better therapeutic effect. Combination magnetic stimulation can strengthen positive sensory input and motor control training to improve the excitability of sensorimotor cortex through rPMS and activate the corresponding brain functional areas to improve neural plasticity through rTMS, which is consistent with the central-peripheral-central (CPC) closed-loop rehabilitation theory proposed by Jia et al. (44). The combination of peripheral and central magnetic stimulation can excite the central and peripheral nervous systems through rTMS from top to bottom and rPMS from bottom to top, completing the integration of central and peripheral interventions, forming a magnetic stimulation closed-loop information feedback, and promoting a long-term enhancement of the main motor cortex on the affected side, thereby helping the recovery of upper limb function after stroke.

Although this study innovatively combines single rTMS, single rPMS, and combined magnetic stimulation to analyze the therapeutic effects and mechanisms of different magnetic stimulation methods on upper limb motor sensory function after stroke, there are still some shortcomings. Firstly, the aim of this study was to compare the differences in efficacy of three types of magnetic stimulation on upper limb dysfunction after stroke. Therefore, no blank control was set in this study. Additionally, no sham stimulation was provided during the magnetic stimulation intervention in the three groups. Future studies could include blank control groups and additional sham stimulation to standardize the experimental design. Secondly, the sample size of this study is relatively small, and the observation period is short, which prevents further tracking of the subsequent effects of treatment. In the future, more patients could be enrolled to obtain further evidence.

## 5 Conclusion

RPMS and rTMS have comparable effects in promoting upper limb motor dysfunction, while combined treatment has better advantages in improving motor function, and rPMS has certain advantages in improving proprioceptive recovery. All three magnetic stimulation methods can promote brain function remodeling after stroke, and the combination therapy can better promote the closed-loop information feedback of magnetic stimulation and promote brain function reorganization through the integration of peripheral and central intervention. Combined magnetic stimulation may be a better choice of magnetic stimulation to repair upper limb dysfunction after stroke.

## Data Availability

The original contributions presented in the study are included in the article/supplementary material, further inquiries can be directed to the corresponding authors.
